# Hemoadsorption in Heart Failure Requiring Mechanical Circulatory Support—A Systematic Review and Meta-Analysis

**DOI:** 10.31083/j.rcm2405137

**Published:** 2023-05-05

**Authors:** Sebastian Freiburger, Tulio Caldonazo, Panagiotis Tasoudis, Gloria Färber, Paul Christian Schulze, Marcus Franz, Torsten Doenst, Hristo Kirov, Mahmoud Diab

**Affiliations:** ^1^Department of Cardiothoracic Surgery, Jena University Hospital-Friedrich Schiller University of Jena, 07747 Jena, Germany; ^2^Division of Cardiothoracic Surgery, University of North Carolina, Chapel Hill, NC 27599, USA; ^3^Department of Internal Medicine I, Jena University Hospital-Friedrich Schiller University of Jena, 07747 Jena, Germany

**Keywords:** Cytosorb®, extracorporeal membrane oxygenation, left ventricular assist device, heart failure

## Abstract

**Background:**

Left ventricular assist devices (LVAD) and extracorporeal membrane oxygenation (ECMO) are well established therapies in heart failure (HF) management. Their use is generally associated with a sudden increase in inflammatory mediators, which are often already elevated in patients with HF prior to device implantation. An exaggerated release of proinflammatory cytokines is associated with organ dysfunction and increased mortality. Hemoadsorption has been shown to reduce inflammatory mediators during cardiopulmonary bypass.

**Objective:**

To investigate the role of hemoadsorption during the management of acute or chronic heart failure with mechanical circulatory support and its impact on survival.

**Methods:**

We systematically searched MEDLINE selecting all studies comparing the use of hemoadsorption during LVAD implantation or veno-arterial (v.a.) ECMO therapy. Records were screened by two different investigators. Reports without a control group and duplicates were excluded.

**Results:**

Our search delivered six studies. One was randomized and five were retrospective studies, of which three were risk-adjusted. During LVAD implantation, one study showed no difference in mortality but higher incidence of respiratory insufficiency in the hemoadsorption group (54% vs 30%, *p* = 0.024) and the other study found higher mortality in the hemoadsorption group (33% vs 0%, *p* = 0.01). During ECMO therapy, three of four studies including the randomized one found no difference in survival or major adverse cardiac events between the hemoadsorption and the control groups. Only one study found lower mortality in the hemoadsorption group (20% vs 60%. *p* = 0.02).

**Conclusions:**

The results of this literature review suggest that the use of hemoadsorption in patients undergoing LVAD implantation might be associated with higher morbidity and mortality. The majority of studies on the use of hemoadsorption during v.a. ECMO therapy showed no effect on mortality or organ dysfunction, while only one small study showed that hemoadsorption was able to reduce mortality. The results are limited by the retrospective nature and the small sample sizes of the majority of the studies included.

## 1. Introduction

Acute and chronic heart failure (HF) affect millions of patients worldwide and both are associated with poor outcome [1]. Regardless of the underlying etiology, HF is associated with both, local and systemic activation of inflammation [2, 3]. There is a prevailing consensus that inflammation has a negative impact on heart failure. The proinflammatory cytokines have been associated with suppression of contractile function of cardiomyocytes, stimulating microvascular inflammation, cardiomyocytes apoptosis, extracellular matrix degradation and cardiac fibrosis [4]. Strategies that interfere with the inflammatory response in HF are effective in reducing myocardial infarct size in response to ischemia/reperfusion injury [5, 6].

Furthermore, mechanical circulatory support devices [e.g., left ventricular assist devices (LVAD), veno- arterial extracorporeal membrane oxygenation (v.a. ECMO), etc.] have been increasingly used in the treatment of both, acute and chronic heart failure and their implantation has been associated with increased inflammation [7]. An exaggerated inflammatory response during cardiopulmonary bypass (CPB) or ECMO, for example, is associated with higher degree of organ dysfunctions and higher mortality [8, 9]. Therefore it appears reasonable to assume that hemoadsorption aiming at reducing cytokines may consecutively dampen the inflammatory response, prevent organ dysfunction and improve survival in heart failure patients [10].

One of the most widely used hemoadsorption devices is the Cytosorb® (Cytosorbens Corporation, Princeton, NJ, USA). Cytosorb® is designed for the extracorporeal reduction of cytokines in the circulating blood, and has been widely used as adjuvant therapy in patients with sepsis. This hemoadsorption device has the ability to rapidly reduce many key cytokines in experimental endotoxemia settings and has been associated with reductions in organ injuries and improvement of survival in animal models [11, 12]. It can be integrated into extracorporeal blood circuits and can successfully absorb mid- molecular weight cytokines, chemotoxins and exotoxins (~5–60 kDa) [10, 13].

Although there are several studies on the use of Cytosorb® in the context of v.a. ECMO therapy or LVAD implantation in patients with heart failure, their results have been inconsistent [14, 15].

Another hemoadsorbtion device (e.g., HA380, Jafron, Zhuhai City, Guangdong, China) has been also designed aiming at reducing the systemic inflammatory response and improving postoperative recovery [16], however its use in patients with heart failure has been limited so far.

The aim of this literature review is to summarize and discuss the results of studies investigating the role of hemoadsorption in heart failure patients including patients with v.a. ECMO or LVAD.

## 2. Methods

Ethical approval of this analysis was not required as no human or animal subjects were involved.

### 2.1 Search Strategy

We performed a comprehensive literature search to identify contemporary studies reporting the use of Cytosorb® in v.a. ECMO and LVAD patients. Searches were run on January 2023 in the Ovid MEDLINE® database. The search strategy is available in **Supplementary Table 1**.

### 2.2 Study Selection and Data Extraction

The study selection followed the Preferred Reporting Items for Systematic Reviews and Meta-Analyses (PRISMA) strategy [17]. After deduplication, records were screened by two independent reviewers (SF and TC). Any discrepancies and disagreements were resolved by a third author (HK). Titles and abstracts were reviewed against pre-defined inclusion and exclusion criteria. Studies were considered for inclusion if they were written in English and reported about heart failure patients that were treated with hemoadsorption (either hemoadsorption during the use of v.a. ECMO or hemoadsorption during LVAD implantation). Animal studies, duplicates, case reports, case series without any control group, commentaries, editorials, expert opinions, conference presentations were excluded.

The full text was pulled for the selected studies for a second round of eligibility screening. References for articles selected were also reviewed for relevant studies not captured by the original search.

The quality of the included studies was assessed using the Newcastle-Ottawa Scale for observational studies (**Supplementary Table 2**) and the Cochrane risk-of-bias tool for randomized trials (**Supplementary Table 3**) [18].

## 3. Results

**Fig. 1.**
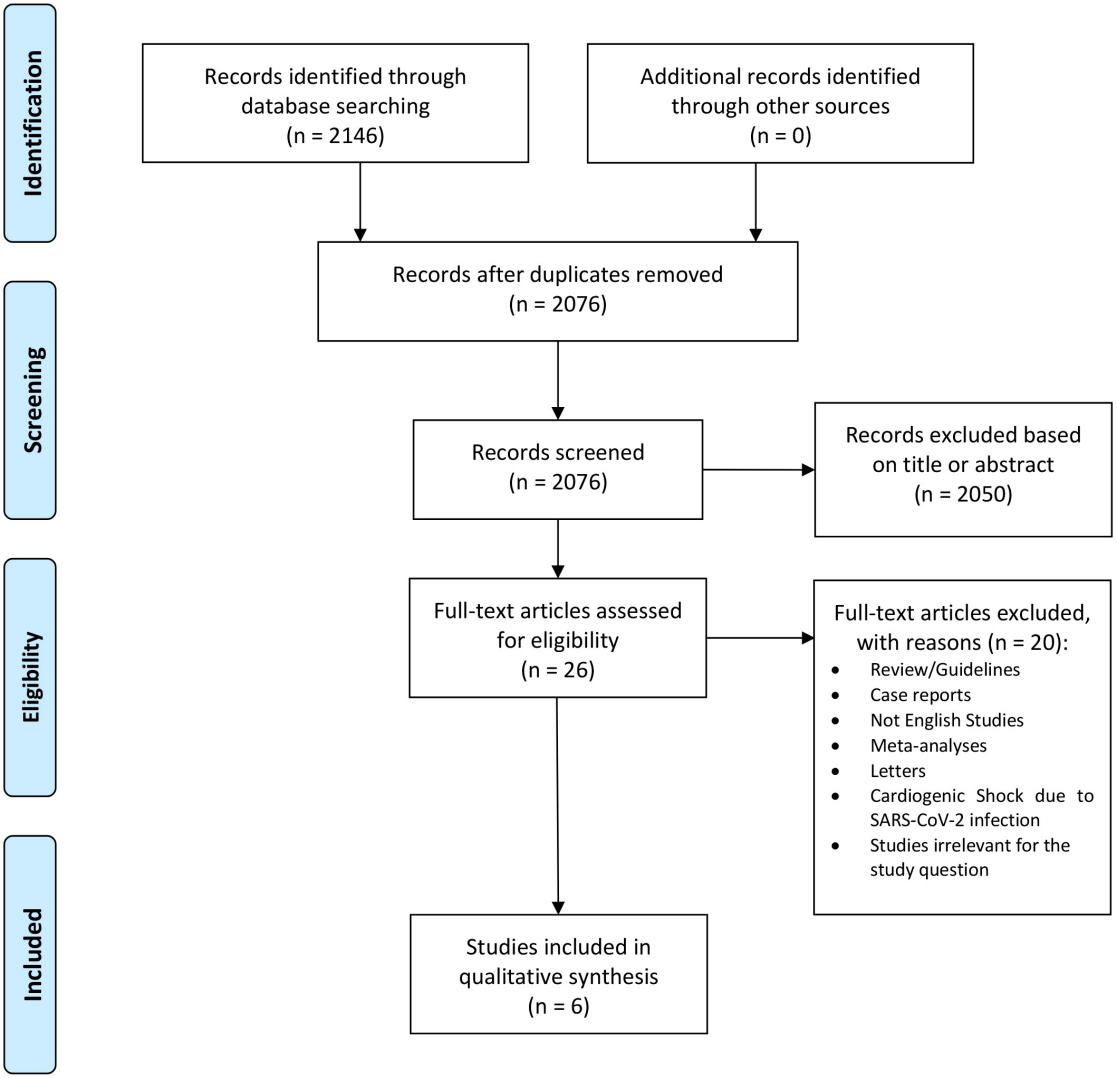
**PRISMA diagram describing the systematic research inclusion/exclusion criteria and the study structure**.

Fig. 1 shows the PRISMA flowchart for study selection [17]. A total number of 2146 studies were retrieved from the systematic search, of which six met the criteria for inclusion in the final analysis.

The six studies were analysed using random-effects model (DerSimonian-Laird) and a two sub-group analysis (ECMO and LVAD studies) were performed. The overall odds ratio was also reported.

**Fig. 2.**
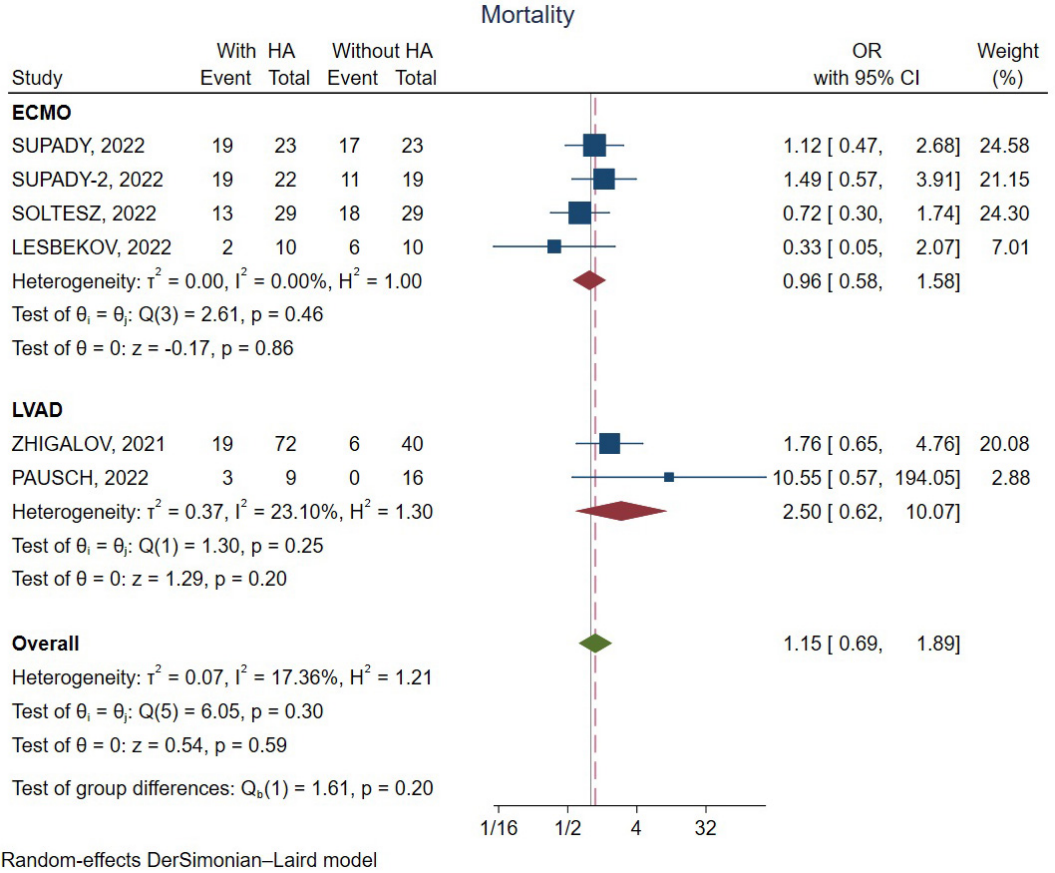
**Forest plot for in-hospital/30-day mortality**. CI, confidence interval; ECMO, extracorporeal membrane oxygenation; HA, hemoadsorption; LVAD, left ventricular assist device; OR, odds ratio.

Fig. 2 shows the forest plot for in-hospital/30-day mortality of the included studies. There was no significant difference between the assist device implantation groups with and without hemoadsorption (OR, odds ratio: 1.15, confidence interval, 95% CI 0.69–1.89, *p* = 0.20).

The data about biomarkers was extremely heterogeneous regarding the type of biomarkers, the kit used, the laboratory references and the metric units.

Table 1 (Ref. [14, 15, 16, 19, 20, 21]) provides the details of the included studies. The included studies were published in the year 2022, one study presented a randomized population [22] and the others were observational cohort studies. The studies originated from Germany, Kazakhstan and Hungary.

**Table 1.** **Studies reporting on the use of hemoadsorption in heart failure patients during LVAD implantation or v.a. ECMO therapy**.

Author	Year	Country	N° of patients	Comparability	Hemoadsorption	Mortality	Biomarkers reduction	Other findings
Zhigalov [19]	2022	Germany	112	PSM	Cytosorb® in CPB during LVAD implantation	No difference	No difference	No difference in MACE, longer ventilation time and more tracheotomy in Cytosorb®
Pausch [16]	2022	Germany	25	No adjustment	Cytosorb® in CPB during LVAD implantation	Higher in Cytosorb® group	No difference	No difference in the need for vasopressors
Lesbekov [15]	2022	Kazakhstan	30	PSM	Cytosorb® or Jafron use in v.a. ECMO	Lower in Cytosorb® and Jafron group	Lower in Cytosorb® and Jafron group	Longer CPB, aortic cross clamp time and ICU stay in Cytosorb®
Soltesz [14]	2022	Hungary	58	No adjustment	Cytosorb® use in v.a. ECMO	No difference	Lower lactate and CRP was higher in Cytosorb® group	Observed vs. expected mortality lower in Cyotosorb® group
Supady [21]	2022	Germany	46	PSM	Cytosorb® use in v.a. ECMO	No difference	No difference	No difference in the need of vasopressor
Supady [20]	2022	Germany	50	RCT	Cytosorb® use in v.a. ECMO	No difference	No difference	No difference in the need for vasopressors

LVAD, left ventricular assist devices; ECMO, extracorporeal membrane oxygenation; PSM, propensity score matching; RCT, randomized clinical trial; CPB, cardiopulmonary bypass; MACE, major adverse cardiovascular events; ICU, intensive care unit; CRP, C-reactive protein.

A total of 321 patients were included in the final analysis, and the number of patients in each study ranged from 25 to 112. The first two studies included 137 heart failure patients, among them 81 received hemoadsorption using Cytosorb® during cardiopulmonary bypass (CBP) required for LVAD implantation.

The study by Zhigalov *et al*. [19] is the one with the largest number of patients. Seventy-two patients were treated with Cytosorb® and compared to 40 propensity- matched patients. There was no difference in the primary endpoint (overall survival). Patients treated with Cytosorb® had more frequently respiratory failure (54% vs 30%, *p* = 0.024), needed more frequently prolonged ventilation for longer than 6 days (50% vs 28%, *p* = 0.035), and required more frequently tracheotomy (32% vs 13, *p* = 0.04) than the control group. The use of hemoadsorption did not show a reduction in white blood cell count (WBC), C- reactive protein (CRP), procalcitonin (PCT), or interleukin 6 (IL-6).

In another study on the use of hemoadsorption during LVAD implantation, Pausch *et al*. [16], included 9 patients who received Cytosorb® and matched them with sixteen patients who did not receive Cytosorb®. Mortality at 30 days was significantly higher in the Cytosorb® group (33% vs 0%, *p* = 0.01). They also found that the use of Cytosorb® was not associated with reduction in vasopressor requirements or increased lactate clearance.

The following four studies included 184 patients with HF who had to be treated with a v.a. ECMO. Among them, in 88 patients hemoadsorption was integrated to v.a. ECMO circuit.

The study, Supady *et al*. [20] randomized 41 patients who were treated with v.a. ECMO after resuscitation into those who received Cytosorb® (n = 22) and those who did not (n = 19). They found no difference in survival, levels of biomarkers, or vasopressor requirement between the two groups.

In a retrospective study by Lesbekov *et al*. [15] 20 patients treated with v.a. ECMO received hemoadsorption either with Cytosorb® (n = 10) or with Jafron (n = 10) and compared them to patients who did not receive any hemoadsorption during v.a. ECMO (the control group). In addition to in-hospital mortality, they evaluated levels of inflammatory markers (IL-1α, IL-6, CRP, Leukocyte, PCT, NT-proBNP, and TNF-α) before, during and after hemoadsorption.They found a significantly higher mortality rate in the control group (60% vs 20%, *p* = 0.02). Both hemoadsorbers showed a significant reduction in IL-6 and PCT compared to the control group. However, almost all inflammatory markers (IL-1α, IL-6, CRP, PCT, and TNF-α) and lactate levels before starting hemoadsorption were significantly higher in the control group compared to the hemoadsorption group.

Soltesz *et al*. [14] included nine patients with v.a. ECMO and Cytosorb® and matched them to 29 patients with v.a. ECMO without Cytosorb®. There was no statistically significant difference in survival. They demonstrated significant reductions in the vasoactive inotropic score, the Sequential Organ Failure Assessment (SOFA) score, plasma levels of lactate and delta CRP, and fewer bleeding complications due to hemoadsorption.

A retrospective propensity- matched study by Supady *et al*. [21] showed no difference in survival or biomarkers between the patients who received Cytosorb® during v.a ECMO therapy (n = 23) compared to those who did not (n = 23).

## 4. Discussion

The results of this literature review suggest that the use of hemoadsorption in patients undergoing LVAD implantation might be associated with higher morbidity and mortality. The majority of studies on the use of hemoadsorption during v.a. ECMO therapy showed no effect on mortality or organ dysfunction, while only one small study showed that hemoadsorption was able to reduce mortality. The results are limited by the retrospective nature and the small sample sizes of the majority of the studies included.

Regardless of the main underlying etiology, excessive release of inflammatory mediators has been linked with poor outcome [4, 23, 24]. Therefore, the extracorporeal removal of cytokines has been proposed as a potential strategy to modulate the immune response and has gained wide acceptance in different clinical scenarios such as sepsis, acute respiratory distress syndrome, cardiac surgery, or heart failure [11, 12, 25].

Cytosorb® is the most widely used hemoadsorption device. It has the ability to rapidly reduce key cytokines in experimental settings of endotoxemia [11] and has been associated with fewer organ injuries and longer survival in animal models [12]. Hundreds of studies, mainly case reports or observational studies, reporting on the impact of Cytosorb® on inflammatory response in different clinical scenarios have been published. The randomized evidence regarding the use of Cytosorb® as adjuvant therapy in sepsis [26, 27] or during cardiopulmonary bypass [28, 29, 30] failed to show a positive effect on clinical outcomes. Importantly, these randomized studies showed that the use of Cytosorb® in these two indications was not associated with any additional adverse events [26, 27, 28, 29, 30].

In one of the studies included in the current literature review, Pausch* et al*. [16], demonstrated higher 30-day mortality in the Cytosorb® group. Despite the limitations in their study, i.e., the small sample, the retrospective nature, and lack of selection criteria for Cytosorb® therapy, the study raises a question regarding the safety of hemoadsorption in certain indications. In HF, there is controversy surrounding the role of inflammation [31]. Inflammation was shown to be protective in mice with pressure overload after aortic banding [32]. In addition, some interventions, such as systemic depletion of macrophages, exacerbated heart failure, suggesting that inflammation has a protective role in HF [33]. The inflammatory response in the failing heart is characterized by induction and activation of a wide range of pleiotropic cytokines and chemokines that modulate phenotype and function of all myocardial cells [4]. Nonselective extracorporeal removal of these inflammatory mediators via hemoadsorption aiming at reduction of this complex and pleiotropic inflammatory response may not be the right therapy.

In a recently published randomized controlled study on patients with COVID-19 with venovenous ECMO, patients were assigned to receive or not receive Cytosorb®. The study demonstrated that mortality was higher in the Cytosorb® group. The authors assumed that since cytokine adsorption using the Cytosorb® device is non-selective, hemoadsorption might have affected concentrations of protective factors as well [34].

The two studies concerning Cytosorb® use in CPB during LVAD implantation performed by Pausch *et al*. [16] and Zhigalov* et al*. [19] were not able to show a significant reduction in the biomarkers after hemoadsorption. A possible explanation for the lack of effect despite the application of Cytosorb® may be the relatively low preoperative level of cytokines in the two studies’ populations. The removal of cytokines by Cytosorb® is concentration dependent. If the concentrations of inflammatory mediators are not high enough, the removal efficacy decreases [12]. One may also argue that the short application time of the Cytosorb® in the CPB during surgery in these two studies may be responsible for the lack of effect on cytokine levels. However, longer application of Cytosorb® (for 42–72 hours) in patients with sepsis did not lead to cytokine-reduction in previous randomized studies [26, 34].

Based on the limited data avialble, the use of Cytosorb® during LVAD-implantation is not only non-beneficial, but it may even be associated with harmful adverse events, as shown by Zhigalov *et al*. [19], or higher mortality, as shown by Pausch *et al*. [16]. Therefore, the current evidence does not justify the use of hemaoadsorption outside clinical trials for patients undergoing LVAD implantation. A randomized study evaluating the efficacy of Cytosorb® in attenuating perioperative changes in IL-6 during LVAD implantation on 60 patients is recruiting (*ClinicalTrials.gov Identifier*: NCT04596813). However, more randomized studies addressing clinically relevant outcome points such as organ dysfunction, mortality, or perioperative hemodynamic measurements are needed.

Two of the four studies, including a randomized study, on the use of hemoadsorption during v.a. ECMO therapy showed no effect of Cytosorb® on cytokine levels, mortality, or organ dysfunction [20, 21]. Only one small study on the use use of Cytosorb® (n = 10) or Jafron (n = 10) during v.a. ECMO therapy was able to show a reduction in inflammatory mediators associated with improved survival in the hemoadsorption group [15]. Randomized studies that failed to identify a reduction of mortality with hemoadsorption for other indications also failed to identify a reduction in cytokine levels despite the use of hemoadsorption [20, 29, 34, 35]. Therefore, it is expected to observe a survival benefit if the hemoadsorption succeeds to reduce inflammatory mediators. However, the study from Soltesz *et al*. [14], included in this current literature review, demonstrated a reduction in biomarker levels associated with the use of Cytosorb® during v.a. ECMO, but without any effect on survival. This lack of effect of hemoadsorption on clinically relevant outcome points despite a detected reduction of cytokine levels was recently observed in the largest randomized study on hemoadsorption in patients undergoing surgery for infective endocarditis, the REMOVE-Trial [30]. Thus, again nonselective extracorporeal removal of inflammatory mediators via hemoadsorption aiming at reduction of the complex and pleiotropic inflammatory response might not be the right therapy. There is a need for individual patient-level meta-analyses on patients with detected reduction compared to those without any reduction of cytokines in response to hemoadsorption, in order to investigate the hypothesis that the reduction of inflammatory mediators is a beneficial therapy in cases with excessive inflammatory response. Such analyses will help us to identify potential groups of patients who may benefit from this innovative therapy.

Irrespective of the undelying potential mechanisms associated with the use of hemoadsorbtion and the extent of its impact on inflammatory parameters in patients with heart failure requiring mechanical circulatory support, the lack of improvement in hard clinical endpoints (survival, organ function) reported in the summarized evidence we present is important as it might influence clinical decision making when considering future treatment. The data from the studies oscillate between centers and the analysis of them implies the intrinsic limitations of observational series, including the risk of potential methodological heterogeneity. That is why there is a need for more adequately powered randomized studies investigating the effect of hemoadsorption in HF patients on outcome. Currently, a single center randomized study (ECMOsorb) investigating the impact of Cytosorb® during v.a. ECMO in patients with cardiogenic shock on hemodynamic changes using the inotropic score as a primary outcome measure is recruiting (ClinicalTrials.gov Identifier: *NCT05027529*). We hope that the results of this study will help in selecting patients who might benefit form such an innovative therapy.

### Limitations

The impact of this review are limited by the retrospective nature of the majority of studies, the lack of adjustment in half of them and the limited number of patients included. Furthermore, patient management among included studies was performed according to individual centre strategy with heterogeneous approaches which may further limit the definitive value of the conclusions.

## 5. Conclusions

The results of this meta-analysis showed that the use of hemaoadsorption during left ventricular assist device implantation or during ECMO therapy was not associated with reduction of 30-day mortality. The results are limited by the retrospective nature and the small sample sizes of the majority of the studies included. The majority of studies on the use of hemoadsorption during v.a. ECMO therapy showed no effect on mortality or organ dysfunction, while only one small study showed that hemoadsorption was able to reduce mortality. The results are limited by the retrospective nature and the small sample sizes of the majority of the studies included.

## Supplementary Material

Supplementary material associated with this article can be found, in the online version, at https://doi.org/10.31083/j.rcm2405137.
