# Supplementary material for: Hemoadsorption in Heart Failure Requiring Mechanical Circulatory Support—A Systematic Review and Meta-Analysis
Source: Rev Cardiovasc Med. 2023 May 5;24(5):137. doi: 10.31083/j.rcm2405137 (PMC11273042; doi:10.31083/j.rcm2405137)
Supplement: Supplementary file 1 [file 2153-8174-24-5-137-s1.zip › Supplementary Material.docx]

**Supplementary Material**

**Supplementary Table 1**: Search strategy for Ovid MEDLINE.

**Supplementary Table 2:** Assessment of risk of bias using the Newcastle-Ottawa Scale.

**Supplementary Table 3**: Assessment of risk of bias using the Cochrane Risk of Bias Tool.

**Supplementary Table 4**: Demographic Data.

**Table 1.** Search strategy for Ovid MEDLINE.

| Search: **(heart failure) AND (hemoadsorption) AND (Extracorporeal membrane oxygenation) AND (Left Ventricular Assist Device) AND (cytosorb) AND (jafron)**  ("heart failure"[MeSH Terms] OR ("heart"[All Fields] AND "failure"[All Fields]) OR "heart failure"[All Fields]) AND "hemoadsorption"[All Fields] AND ("extracorporeal membrane oxygenation"[MeSH Terms] OR ("extracorporeal"[All Fields] AND "membrane"[All Fields] AND "oxygenation"[All Fields]) OR "extracorporeal membrane oxygenation"[All Fields]) AND ("Left"[All Fields] AND ("heart assist devices"[MeSH Terms] OR ("heart assist"[All Fields] AND "devices"[All Fields]) OR "heart assist devices"[All Fields] OR ("ventricular"[All Fields] AND "assist"[All Fields] AND "device"[All Fields]) OR "ventricular assist device"[All Fields])) AND ("cytosorb"[All Fields] OR "cytosorbents"[All Fields]) AND "jafron"[All Fields] |
| --- |
| **Translations** |
| **heart failure:** "heart failure"[MeSH Terms] OR ("heart"[All Fields] AND "failure"[All Fields]) OR "heart failure"[All Fields]  **Extracorporeal membrane oxygenation:** "extracorporeal membrane oxygenation"[MeSH Terms] OR ("extracorporeal"[All Fields] AND "membrane"[All Fields] AND "oxygenation"[All Fields]) OR "extracorporeal membrane oxygenation"[All Fields]  **Ventricular Assist Device:** "heart-assist devices"[MeSH Terms] OR ("heart-assist"[All Fields] AND "devices"[All Fields]) OR "heart-assist devices"[All Fields] OR ("ventricular"[All Fields] AND "assist"[All Fields] AND "device"[All Fields]) OR "ventricular assist device"[All Fields]  **cytosorb:** "cytosorb"[All Fields] OR "cytosorbents"[All Fields] |

**Table 2.** Assessment of risk of bias using the Newcastle-Ottawa Scale.

| AUTHOR | Year of publication | SELECTION | COMPARABILITY | OUTCOME/ EXPOSURE |
| --- | --- | --- | --- | --- |
| Zhigalov [21] | 2022 | *** | ** | ** |
| Lesbekov [23] | 2022 | *** | * | *** |
| Soltesz [24] | 2022 | *** | ** | *** |
| Supady [25] | 2022 | *** | ** | *** |
| Pausch [22] | 2022 | *** | * | *** |

**Table 3.** Assessment of risk of bias using the Cochrane Risk of Bias Tool.

| **Author** | **Year of publication** | **RANDOM**  **SEQUENCE**  **GENERATION** | **ALLOCATION**  **CONCEALMENT** | **BLINDING OF**  **PARTICIPANTS** | **BLINDING OF**  **OUTCOME**  **ASSESSMENT** | **INCOMPLETE**  **OUTCOME DATA** | **SELECTIVE**  **REPORTING** | **OTHER SOURCES**  **OF BIAS** |
| --- | --- | --- | --- | --- | --- | --- | --- | --- |
| Supady [26] | 2022 | + | + | - | - | + | + | + |
|  | | | | + | Low Risk | | | |
|  |  |  |  | ? | Uncertain | | | |
|  |  |  |  | - | High Risk | | | |

**Table 4** Demographic Data.

|  | Zhigalov | | Pausch | | Lesbekov | | | Soltesz | | Supady | | Supady | |
| --- | --- | --- | --- | --- | --- | --- | --- | --- | --- | --- | --- | --- | --- |
|  | **Cytosorb** | **Control** | **Cytosorb** | **Control** | **Cytosorb** | **Jafron** | **Control** | **Cytosorb** | **Control** | **Cytosorb** | **Control** | **Cytosorb** | **Control** |
| Age, years | 56.3 ± 12.3 | 57.9 ± 9.5 | 57 (50–62) | 56 (51–62) | 48.6 ± 17.6 | 47.5 ± 13.1 | 59.1 ± 13.7 | 51 ± 15 | 55 ± 14 | 53.3 ± 18.2 | 52.7 ± 17.7 | n.a. | n.a. |
| Male % | 84,7 | 77,5 | 8 (88.9) | 13 (81.3) | 3 (30%) | 6 (60%) | 2 (20%) | 72,4% | 73% | 18 (78%) | 17 (74%) | n.a. | n.a. |
| BMI, kg/m² | 24.6 (22.2– 28.4) | 25.6 (23.5– 27.9) | 29.8 ± 5.7 | 27.3 ± 4.9 | 25.4 ± 4.2 | 29.2 ± 4.6 | 27.5 ± 4.0 | 27.8 ± 4.6 | 28.2 ± 5.5 | 28.1 ± 5.8 | 25.4 ± 4.4 | n.a. | n.a. |
| **Comorbidities, n (%)** | | | | | | | | | | | | | |
| Hypertension | 45 (62.5) | 28 (70.0) | 3 (33.3) | 6 (37.5) | n.a. | n.a. | n.a. | 10 (34.5%) | 13 (44.8%) | 8 (34.8%) | 7 (30.4%) | n.a. | n.a. |
| Coronary artery disease | 36 (50.0) | 22 (55.0) | n.a. | n.a. | n.a. | n.a. | n.a. | 11 (37.9%) | 14 (48.3%) | n.a. | n.a. | n.a. | n.a. |
| Hyperlipidemia | 26 (36.1) | 17 (42.5) | n.a. | n.a. | n.a. | n.a. | n.a. | n.a. | n.a. | n.a. | n.a. | n.a. | n.a. |
| Smoking history | 39 (54.2) | 26 (65.0) | n.a. | n.a. | n.a. | n.a. | n.a. | n.a. | n.a. | 6 (26.1%) | 6 (26.1%) | n.a. | n.a. |
| Atrial fibrilation | 28 (38.9) | 20 (50.0) | 3 (33.3) | 12 (75.0) | n.a. | n.a. | n.a. | n.a. | n.a. | n.a. | n.a. | n.a. | n.a. |
| Diabetes | 24 (33.3) | 10 (25.0) | 2 (22.2) | 3 (18.8) | n.a. | n.a. | n.a. | 8 (27.6%) | 7 (24.1%) | 6 (26.1%) | 5 (21.7%) | n.a. | n.a. |
| peripherial artery disease | 12 (16.7) | 4 (10.0) | n.a. | n.a. | n.a. | n.a. | n.a. | 0 (0%) | 3 (10.3%) | n.a. | n.a. | n.a. | n.a. |
| Chronic obstructive pulmonary disease | 13 (18.1) | 7 (17.5) | 1 (11.1) | 2 (12.5) | n.a. | n.a. | n.a. | 6 (20.7%) | 3 (10.3%) | 5 (21.7%) | 2 (8.7%) | n.a. | n.a. |
| Stroke | 6 (8.33) | 2 (5.00) | 0 (0.0) | 3 (18.8) | n.a. | n.a. | n.a. | 2 (6.9%) | 3 (10.3%) | n.a. | n.a. | n.a. | n.a. |
| Infection | 19 (26.4) | 12 (30.0) | n.a. | n.a. | n.a. | n.a. | n.a. | n.a. | n.a. | n.a. | n.a. | n.a. | n.a. |
| Myocardial infarktion | 32 (44.4) | 18 (45.0) | n.a. | n.a. | n.a. | n.a. | n.a. | n.a. | n.a. | 6 (26.1%) | 4 (17.4%) | n.a. | n.a. |
| Percutaneus cardiac intervention | 31 (43.1) | 12 (30.0) | n.a. | n.a. | n.a. | n.a. | n.a. | n.a. | n.a. | n.a. | n.a. | n.a. | n.a. |
| Cardiac resynchronisation therapy | 28 (38.9) | 14 (35.0) | n.a. | n.a. | n.a. | n.a. | n.a. | n.a. | n.a. | n.a. | n.a. | n.a. | n.a. |
| Implanted cardioverter defibrillator | 38 (52.8) | 26 (65.0) | n.a. | n.a. | n.a. | n.a. | n.a. | n.a. | n.a. | n.a. | n.a. | n.a. | n.a. |
| Pulmonary hypertension | 34 (47.2) | 19 (47.5) | n.a. | n.a. | n.a. | n.a. | n.a. | n.a. | n.a. | n.a. | n.a. | n.a. | n.a. |
| Preoperativ pericardial effusion | 39 (54.2) | 26 (65.0) | n.a. | n.a. | n.a. | n.a. | n.a. | n.a. | n.a. | n.a. | n.a. | n.a. | n.a. |
| **Primary diagnosis** | | | | | | | | | | | | | |
| Ischemic cardiomyopathy | 33 (45.8) | 17 (42.5) | 4 (44.4) | 6 (37.5) | n.a. | n.a. | n.a. | n.a. | n.a. | n.a. | n.a. | n.a. | n.a. |
| Dilated cardiomyopathy | 36 (50.0) | 21 (52.5) | n.a. | n.a. | n.a. | n.a. | n.a. | n.a. | n.a. | n.a. | n.a. | n.a. | n.a. |
| Toxic cardiomyopathy | 2 (2.78) | 1 (2.50) | n.a. | n.a. | n.a. | n.a. | n.a. | n.a. | n.a. | n.a. | n.a. | n.a. | n.a. |
| Other indiaction | 1 (1.39) | 1 (2.50) | n.a. | n.a. | n.a. | n.a. | n.a. | n.a. | n.a. | n.a. | n.a. | n.a. | n.a. |
| Cardiorespiratory conditions | | | | | | | | | | | | | |
| Mechanical ventilation | 17 (23.6) | 7 (17.5) | n.a. | n.a. | n.a. | n.a. | n.a. | n.a. | n.a. | n.a. | n.a. | n.a. | n.a. |
| Intra- aortic balloon pump | 4 (5.56) | 4 (10) | n.a. | n.a. | n.a. | n.a. | n.a. | n.a. | n.a. | n.a. | n.a. | n.a. | n.a. |
| ECLS | 14 (19.4) | 9 (22.5) | 6 (66.7) | 12 (75.0) | n.a. | n.a. | n.a. | n.a. | n.a. | n.a. | n.a. | n.a. | n.a. |
| Ejection fraction % | 16.5 (15.0– 21.2) | 15.0 (10.0– 22.0) | 17.5 ± 5.6 | 19.5 ± 6.6 | n.a. | n.a. | n.a. | n.a. | n.a. | n.a. | n.a. | n.a. | n.a. |
| Duration of operation | 174 (149– 234) | 188 (163– 236) | 293 (238–330) | 250 (216–293) | n.a. | n.a. | n.a. | n.a. | n.a. | n.a. | n.a. | n.a. | n.a. |
| Cardio pulmonary bypass time | 72.5 (61.8– 99.5) | 73.0 (65.8– 98.5) | 131 (101–151) | 109 (98–164) | 200.8 ± 33.7 | 175.75 ± 80.1 | 111.4 ± 36.3 | n.a. | n.a. | n.a. | n.a. | n.a. | n.a. |
| Isolated procedure | 59 (81.9) | 36 (90.0) | n.a. | n.a. | n.a. | n.a. | n.a. | n.a. | n.a. | n.a. | n.a. | n.a. | n.a. |
| APACHE II | n.a. | n.a. | n.a. | n.a. | 12.2 ± 1.9 | 12 ± 2.6 | 8.46 ± 5.4 | 31.1 ± 5.1 | 30.0 ± 5.5 | n.a. | n.a. | n.a. | n.a. |
| EuroSCORE II | n.a. | n.a. | n.a. | n.a. | 14.3 ± 3.9 | 16.1 ± 2.1 | 10.1 ± 2.3 | n.a. | n.a. | n.a. | n.a. | n.a. | n.a. |
| **Laborato Pre intervention** | | | | | | | | | | | | | |
| WBC, x10^9^/L | 10,9± 5,08 | 10.7 ± 4.16 | 13 (8–20) | 12 (9–14) | n.a. | n.a. | n.a. | 14.45 ± 9.57 | 11.64 ± 4.28 | n.a. | n.a. | n.a. | n.a. |
| CrP, mg/L | 6.97 ± 5.53 | 8.76 ± 8.84 | 66 (51–101) | 45 (27–82) | n.a. | n.a. | n.a. | 66.57 ± 82.23 | 31.57 ± 43.25 | 12.11 ± 23.4 | 43.4 ± 99.2 | n.a. | n.a. |
| Creatinin, mg/dl | 1.47 ± 0.45 | 1.13 ± 0.50 | 2.3 (1.4–2.3) | 2.0 (1.4–2.6) | n.a. | n.a. | n.a. | n.a. | n.a. | n.a. | n.a. | n.a. | n.a. |
| BUN, mg/dl | 0.34 ± 0.16 | 0.26 ± 0.12 | n.a. | n.a. | n.a. | n.a. | n.a. | n.a. | n.a. | n.a. | n.a. | n.a. | n.a. |
| Total bilirubin, | 1.63 ± 1.72 mmol/l | 1.18 ± 0.75 mmol/l | 2.5 (1.1–3.7) mg/dL | 1.4 (0.8–1.6) mg/dL | n.a. | n.a. | n.a. | n.a. | n.a. | n.a. | n.a. | n.a. | n.a. |
| ALT, U/L | 161.8 ± 345.4 | 243.1 ± 586.9 | 118 (106–141) | 116 (30–584) | n.a. | n.a. | n.a. | n.a. | n.a. | n.a. | n.a. | n.a. | n.a. |
| LDH, U/L | 427.1 ± 298.5 | 482.4 ± 432.2 | n.a. | n.a. | n.a. | n.a. | n.a. | n.a. | n.a. | n.a. | n.a. | n.a. | n.a. |
| PCT | 1.44 ± 3.55 | 1.03 ± 2.04 | n.a. | n.a. | n.a. | n.a. | n.a. | n.a. | n.a. | 0.98 ± 1.17 | 2.69 ± 4.20 | n.a. | n.a. |
| IL-6 | 67.7 ± 48.85 | 39.6 ± 37.1 | n.a. | n.a. | n.a. | n.a. | n.a. | n.a. | n.a. |  |  | n.a. | n.a. |
| Laktat mmol/L | n.a. | n.a. | 1.7 (1.3–2.1) | 1.0 (0.7–1.2) | n.a. | n.a. | n.a. | 6.56 ± 4.96 | 6.90 ± 4.12 | 11.20 ± 6.96 | 11.59 ± 5.04 | n.a. | n.a. |
| **Laboratoy Post intervention** | | | | | | | | | | | | | |
| WBC, x10^9^/L | 13.0 ± 5.10 | 13.0 ± 4.74 | n.a. | n.a. | n.a. | n.a. | n.a. | n.a. | n.a. | n.a. | n.a. | n.a. | n.a. |
| CrP, mg/L | 11.6 ± 5.74 | 11.5 ± 7.92 | n.a. | n.a. | n.a. | n.a. | n.a. | 116.69 ± 55.33 | 140.05 ± 86.72 | 221.29 ± 66.0 | 141.8 ± 129.6 | n.a. | n.a. |
| Creatinin, mg/dl | 1.46 ± 0.66 | 1.44 ± 0.94 | n.a. | n.a. | n.a. | n.a. | n.a. | n.a. | n.a. | n.a. | n.a. | n.a. | n.a. |
| BUN, mg/dl | 0.35 ± 0.32 | 0.34 ± 0.32 | n.a. | n.a. | n.a. | n.a. | n.a. | n.a. | n.a. | n.a. | n.a. | n.a. | n.a. |
| Total bilirubin, mmol/l | 2.50 ± 3.73 | 1.63 ± 2.30 | n.a. | n.a. | n.a. | n.a. | n.a. | n.a. | n.a. | n.a. | n.a. | n.a. | n.a. |
| ALT, U/L | 126.4 ± 349.3 | 93.8 ± 223.0 | n.a. | n.a. | n.a. | n.a. | n.a. | n.a. | n.a. | n.a. | n.a. | n.a. | n.a. |
| LDH, U/L | 465.5 ± 700.0 | 416.8 ± 356.5 | n.a. | n.a. | n.a. | n.a. | n.a. | n.a. | n.a. | n.a. | n.a. | n.a. | n.a. |
| PCT | 4.66 ± 11.5 | 1.59 ± 2.31 | n.a. | n.a. | n.a. | n.a. | n.a. | n.a. | n.a. | 34.67 ± 56.45 | 8.17 ± 12.54 | n.a. | n.a. |
| IL-6 | 82.9 ± 64.7 | 35.0 ± 34.7 | n.a. | n.a. | n.a. | n.a. | n.a. | n.a. | n.a. | n.a. | n.a. | n.a. | n.a. |
| lactat mmol/L | n.a. | n.a. | n.a. | n.a. | n.a. | n.a. | n.a. | 1.57 ± 0.96 | 2.11 ± 0.77 | 2.66 ± 2.35 | 2.58 ± 3.46 | n.a. | n.a. |

Abbreviations: ALT, alanin transaminase; BUN, blood urea nitrogen; CRP, C- reactive protein; IL- 6, interleukin 6; LDH, Lactate dehydrogenase; PCT, procalcitonin; WBC, white blood cell count; n.a. not available .
